# Supplementary material for: Protocol for individual participant data meta-analysis of randomised controlled trials of patients with psychosis to investigate treatment effect modifiers for CBT versus treatment as usual or other psychosocial interventions
Source: BMJ Open. 2021 May 28;11(5):e035062. doi: 10.1136/bmjopen-2019-035062 (PMC8166625; doi:10.1136/bmjopen-2019-035062)
Supplement: Author's manuscript [file bmjopen-2019-035062.draft_revisions.pdf]

BMJ Open is committed to open peer review. As part of this commitment we make the peer review history of every article we publish publicly available.

When an article is published we post the peer reviewers' comments and the authors' responses online. We also post the versions of the paper that were used during peer review. These are the versions that the peer review comments apply to.

The versions of the paper that follow are the versions that were submitted during the peer review process. They are not the versions of record or the final published versions. They should not be cited or distributed as the published version of this manuscript.

BMJ Open is an open access journal and the full, final, typeset and author-corrected version of record of the manuscript is available on our site with no access controls, subscription charges or pay-per-view fees (http://bmjopen.bmj.com).

If you have any questions on BMJ Open's open peer review process please email info.bmjopen@bmj.com

BMJ Open

For whom is Cognitive Behavioural Therapy (CBT) for psychosis most effective? Protocol for an IPD meta-analysis of randomised control trials comparing CBT versus standard care and other psychosocial interventions (Cognitive Behaviour Therapy for Psychosis: Individual Modifiers of Patient Response to Treatment)

Journal:	*BMJ Open*
Manuscript ID	bmjopen-2019-035062
Article Type:	Protocol
Date Submitted by the Author:	17-Oct-2019

Complete List of Authors:	Sudell, Maria; University of Liverpool, Department of Biostatistics, Institute of Translational Medicine<br>Tudur-Smith, Catrin; University of Liverpool, Biostatistics<br>Liao, Xiaomeng; University of Liverpool, Department of Biostatistics, Institute of Translational Medicine<br>Longden, Eleanor; Greater Manchester West Mental Health NHS Foundation Trust<br>Dunn, Graham; University of Manchester, Health Methodology Research<br>Kendall, Tim; National Collaborating Centre for Mental Health<br>Emsley, Richard; King’s College London, Department of Biostatistics and Health Informatics, Institute of Psychiatry, Psychology and Neuroscience<br>Morrison, Anthony; University of Manchester<br>Varese, Filippo; Manchester Academic Health Science Centre,

Keywords:	IPD-MA, Treatment Modifier, CBT, Psychosis

SCHOLARONE™
Manuscripts

I, the Submitting Author has the right to grant and does grant on behalf of all authors of the Work (as defined in the below author licence), an exclusive licence and/or a non-exclusive licence for contributions from authors who are: i) UK Crown employees; ii) where BMJ has agreed a CC-BY licence shall apply, and/or iii) in accordance with the terms applicable for US Federal Government officers or employees acting as part of their official duties; on a worldwide, perpetual, irrevocable, royalty-free basis to BMJ Publishing Group Ltd ("BMJ") its licensees and where the relevant Journal is co-owned by BMJ to the co-owners of the Journal, to publish the Work in this journal and any other BMJ products and to exploit all rights, as set out in our licence.

The Submitting Author accepts and understands that any supply made under these terms is made by BMJ to the Submitting Author unless you are acting as an employee on behalf of your employer or a postgraduate student of an affiliated institution which is paying any applicable article publishing charge ("APC") for Open Access articles. Where the Submitting Author wishes to make the Work available on an Open Access basis (and intends to pay the relevant APC), the terms of reuse of such Open Access shall be governed by a Creative Commons licence – details of these licences and which Creative Commons licence will apply to this Work are set out in our licence referred to above.

Other than as permitted in any relevant BMJ Author's Self Archiving Policies, I confirm this Work has not been accepted for publication elsewhere, is not being considered for publication elsewhere and does not duplicate material already published. I confirm all authors consent to publication of this Work and authorise the granting of this licence.

For peer review only

For whom is Cognitive Behavioural Therapy (CBT) for psychosis most effective? Protocol for an IPD meta-analysis of randomised control trials comparing CBT versus standard care and other psychosocial interventions (Cognitive Behaviour Therapy for Psychosis: Individual Modifiers of Patient Response to Treatment)

Maria Sudell[1,a*], Catrin Tudur Smith[1,b], Xiaomeng Liao[1,c], Eleanor Longden[3,d], Graham Dunn[4], Tim Kendall[5, 6,e], Richard Emsley[7,f] & Anthony P. Morrison[2,3,g] Filippo Varese[2,3,h]

[1]Institute of Translational Medicine, University of Liverpool; [2]School of Health Sciences, Division of Psychology and Mental Health, Faculty of Biology Medicine and Health, University of Manchester; [3]Greater Manchester Mental Health NHS Mental Health Foundation Trust; [4]Institute of Population Health, University of Manchester; [5]National Collaborating Centre for Mental Health; [6]Sheffield Health and Social Care NHS Foundation Trust; [7]Department of Biostatistics & Health Informatics, Kings College London; [a]mesudell@liverpool.ac.uk; [b]cat1@liverpool.ac.uk; [c]Xiaomeng.Liao@liverpool.ac.uk; [d]eleanorlongden@googlemail.com; [e]tim2.kendall@virgin.net; [f]richard.emsley@kcl.ac.uk; [g]Anthony.P.Morrison@manchester.ac.uk ; [h]filippo.varese@manchester.ac.uk;

**Corresponding author*

University of Manchester
Division of Psychology and Mental Health
School of Health Sciences
Zochonis Building, 2nd floor; room 2.40
Manchester
M13 9PL

For peer review only

Article Summary

Introduction: Aggregate data meta-analyses have shown heterogeneous treatment effects for Cognitive Behavioural Therapy (CBT) for patients with schizophrenia-spectrum diagnoses. This heterogeneity could stem from specific intervention or patient characteristics that could influence the clinical effectiveness of CBT, termed treatment effect modifiers. This Individual Participant Data Meta-Analysis (IPD-MA) will investigate a range of potential treatment effect modifiers of the efficacy CBT. **Methods and analysis**: We will perform a systematic review and meta-analysis of studies investigating CBT vs treatment as usual (TAU), or CBT vs other psychosocial interventions, for patients with schizophrenia-spectrum diagnoses. The Cochrane Central Register of Controlled Trials (CENTRAL), PubMed, EMBASE and the online clinical trials registers of the US government, European Union, World Health Organization and Current Controlled Trials Ltd will be searched. Two researchers will screen titles and abstracts identified by the search. Individual participant data will be requested for any eligible study, for the primary outcome (overall psychotic symptoms), secondary outcomes and treatment effect modifiers. Data will be checked and recoded according to an established Statistical Analysis Plan. One and two-stage random effects meta-analyses investigating potential treatment effect modifiers will be conducted. A list of potential treatment effect modifiers for CBT will be produced, motivating future research into particular modifiers. **Ethics and dissemination:** This study does not require ethical approval as it is based on data from existing studies, although best ethical practice for secondary analysis of clinical data will be followed. The findings will be submitted for publication in peer-reviewed journals, and promoted to relevant stakeholders.

Prospero registration number 15/187/05

Strengths and Limitations of study

- This will be the first published IPD-MA to investigate treatment effect modifiers for CBT for patients with schizophrenia-spectrum diagnoses
- The review will consider the efficacy of CBTp across multiple outcomes of interest in addition to psychotic symptoms severity

For peer review only

- The search will be conducted without geographical, language or time restrictions
- A potential limitation of this study will be the degree of heterogeneity between studies in recorded measures and scales employed.

Introduction

Cognitive behavioural therapy (CBT) is a recommended intervention for the treatment and management of psychosis and schizophrenia [2]. Aggregate Data Meta-Analyses (AD-MA) suggest that CBT for psychosis has modest but considerably heterogeneous treatment effects (for example [3, 4]). This inconsistency partly stems from inter-trial variation in several key methodological characteristics of the existing Randomised Controlled Trials (RCT; for example, blinding/masking of outcome assessments [3, 4]) but could also reflect the impact of unaccounted clinical heterogeneity i.e. specific intervention and patient characteristics that can potentially influence the clinical effectiveness of CBT. For instance, previous trials differed widely in terms of intervention characteristics (e.g. number of treatment sessions, treatment duration, use of manualised interventions), patients' baseline severity of psychotic and other comorbid symptoms, their demographic characteristics (e.g. age, gender and ethnic origin), and illness duration. The identification of moderators of treatment response and/or sub-groups of patients who may particularly benefit from CBT would allow optimisation of treatment delivery, with significant implications in terms of improved clinical effectiveness, cost savings and maximisation of patients' informed choice of treatment.

The impact of these potential treatment effect modifiers, however, remains unaccounted for (or at best poorly estimated) in AD-MA due to their reliance of the reporting quality of primary studies and the limited statistical power of "standard" meta-analytic methods for testing treatment effect moderators [5]. Additional primary research would be costly and impractical given the large sample size required. The only approach suited for this type of research is Individual Patient Data Meta-Analysis (IPD-MA), a research synthesis method which summarises the evidence on a particular clinical question by considering individual participant level rather than aggregate level data from multiple related studies. IPD-MA allow (1) greater ability to examine the impact of multiple individual-level and study-level

For peer review only

factors (and their combination) on the treatment effects considered, (2) standardisation of statistical methods used across studies, and (3) the potential of reduced risk of bias e.g. due to selective reporting of outcomes compared to conventional AD-MA [1, 6-8]. This article is the protocol for an Individual Patient Data Meta-Analysis to assess the impact of treatment effect modifiers of CBT.

Objectives of IPD-MA

This research will identify treatment effect modifiers of CBT in people with diagnoses in the schizophrenia-spectrum using IPD-MA methods to combine data from multiple RCTs.

Treatment comparisons

Consistent with the analytic approaches in recent AD-MA [13, 14] our IPD-MA will distinguish between "pure" CBT interventions as defined by NICE [2] and "CBT+" interventions, where CBT+ is defined as a CBT treatment packages incorporating significant elements of other, distinct psychosocial intervention approaches (e.g. mindfulness, motivational interviewing, family intervention) alongside core CBT elements. CBT and CBT+ will be compared to Treatment As Usual (TAU) or other psychosocial interventions in individuals with a diagnosis of schizophrenia-spectrum disorders. TAU is defined as the level of care service users would routinely receive had they not been involved in the trial, other psychosocial interventions is defined as TAU supplemented by additional psychological or social interventions e.g. family therapy. Although all trials of CBT against these comparators will be eligible, these will be synthetized and contrasted in separate analyses for: (1) CBT vs TAU, (2) CBT vs other psychosocial interventions, (3) CBT+ vs TAU, (4) CBT+ vs other psychosocial interventions.

Treatment Effect Modifiers

The selection of treatment effect modifiers was informed by (1) knowledge of variables examined in previous and on-going RCTs by members of our team and the collaborators in the CBTp: IMPART Consortium; (2) the findings of studies which examined predictors of

For peer review only

outcomes in previous RCTs [e.g. 9, 10-12], and (3) consultation meetings with service-users with psychosis and clinical psychologists and CBT therapists working with clients with psychosis in secondary care settings in the UK. Secondary outcomes were selected based on (1) outcomes often targeted in CBT for psychosis, (2) measures identified as valuable in our PPI consultations

The treatment effect modifiers shown in Table 1 will be investigated for both the primary and secondary outcomes. Treatment effect modifiers have been separated into three main groups: (1) Participant Demographic Characteristics, (2) Participant's Clinical (3) Specific Intervention Characteristics

	Treatment effect Modifier
Participant's Demographic Characteristics	Age at entry to trial
	Gender
	Ethnicity
Participant's Clinical Characteristics	Effect of specific diagnostic sub-groups
	Phase of illness (first episode psychosis / multiple episode psychosis)
	Illness Duration
	Duration of untreated psychosis
	Initial Severity of Psychotic Symptoms (measured by baseline PANSS scores)
	Initial severity of comorbid affective symptoms (measured by baseline Anxiety scores)

For peer review only

	Initial severity of comorbid affective symptoms (measured by baseline Depression scores)
	Dosage equivalence of baseline anti-psychotic medication(s) [15]
	Number of anti-psychotic medications received at baseline)
Specific Intervention Characteristics	time period over which treatment was delivered*
	number of therapy sessions offered in the study*
	number of therapy sessions attended by the individual
	Minimum study required level of therapist's training and competence*;
	Measures of therapeutic alliance;
	Use of manualised interventions*;
	Use of formulation-based interventions*;
	Indicator for whether the intervention was designed to target the outcome under scrutiny*;
	Individual versus group interventions*
	Length of study follow up

*Table 1: Treatment effect modifiers examined in this IPD-MA. Note: treatment effect modifiers marked with * are study level variables, the remaining are individual level variables*

For peer review only

Primary outcome

The primary objective is to identify treatment effect modifiers for CBT or CBT+ vs TAU or other psychosocial interventions on overall psychotic symptoms severity as measured by PANSS scores [16, 17] in patients with schizophrenia-spectrum diagnoses.

Secondary outcomes

Treatment effect modifiers for CBT or CBT+ vs TAU or other psychosocial interventions will be examined for the secondary outcomes listed in Table 2. For some secondary outcomes, multiple sub-outcomes will be examined, such as Change in severity of affective symptoms, which is examined both for anxiety and depression. Adverse event information will be sought specifically for the 4 sub-outcomes mentioned, but additional adverse event information will also be tabulated.

**Minimum clinical important differences (MCIDs) in PANSS scores**
$\geq$ 11 points
$\geq$ 15 points
**Change in specific symptom clusters**
Positive psychopathology
Negative psychopathology
General psychopathology
**Change in specific symptoms often targeted in CBT for psychosis**
Hallucinations severity
Delusions severity
Hallucinations associated subjective distress
Delusions associated subjective distress
Paranoia severity
**Change in severity of affective symptoms**
Anxiety
Depression

For peer review only

**Subjectively defined recovery**	
**Quality of Life**	
**Social and Occupational functioning**	
**Early Treatment Discontinuation**	
**Adverse effects**	
	Deaths
	Attempts at suicide
	Suicide ideation
	Serious violent incidents
**Hospital readmissions**	

Table 2: Secondary outcomes

Methods

Protocol

This evidence synthesis will follow state-of-the-art guidelines for IPD-MA, and our outputs will comply as a minimum with the PRISMA statement for the reporting of IPD-MA [1]. The search strategy of our IPD-MA builds on the protocol and database searches carried out as part of a recent AD-MA carried out by members of our team [13, 14]. Our study selection criteria are consistent with those employed in this recent AD-MA. Similarly, our literature searches will update those carried out as part of this review to identify any RCTs that have become available since the date of search.

Search Strategy

We will update the searches already conducted as part of a recent meta-analysis of aggregated data [13, 14] to identify any trials that might have been become available for research synthesis since the date of last search. Database searches will be conducted on the Cochrane Central Register of Controlled Trials (CENTRAL), PubMed, EMBASE and the online clinical trials registers of the US government, European Union, World Health Organization and Current Controlled Trials Ltd.

For peer review only

In line with the protocol of the AD-MA carried out by members of our team [13, 14], titles, abstracts and keywords will be searched in the publication databases using the adaptations of following generic strategy: (schizo$ [exp. schizophrenia + psychosis + schizoaffective]) AND (trial [exp. RCT +controlled trial + clinical trial]) AND (cbt [exp. cognitive therapy + behaviour therapy +psychotherapy]).

Selection of Studies

The project's PI and another member of the research team will screen titles and abstracts for relevance, and subsequently assess eligibility by examining the full-text reports against the above-mentioned criteria. When required, additional information to ascertain eligibility will be requested from the RCTs' authors and through the examination of treatment manual when available (e.g. to ascertain that interventions complied with NICE operational definitions of CBT). Discrepancies in selection decisions will be discussed, and arbitration by other members of the research team sought to achieve consensus. We will include studies based on the following eligibility criteria:

Participants

Trials where > 50% of participants have diagnoses in the schizophrenia-spectrum (schizophrenia, schizoaffective disorder or early psychosis) will be eligible. Trials where > 50% participants have an established diagnosis of bipolar disorder, intellectual disability, psychosis secondary to a general medical condition or organic pathology, or a primary diagnosis of substance-induced psychosis will be excluded. No restriction will be placed on participants' age, ethnicity, illness severity and illness duration.

Treatment Comparisons

Included studies must compare treatments that fit into one or more of the four stated treatment categories (CBT, CBT+, TAU, other psychosocial interventions). Study interventions will be classified into one of these four categories by clinicians involved with this project.

For peer review only

Outcomes

The study must provide data for one or more of the stated primary or secondary outcomes. Data can be recorded on any comparable scale, at any time-point. Data will also be sought for the treatment effect modifiers listed in Table 1.

Study Designs

Parallel single-blind or open controlled trials with at least two arms utilizing random allocation to treatment will be considered for inclusion. Single arm or cross-over studies will not be eligible for inclusion in the review. Studies employing other research designs (case series, cohort analyses) will not be eligible.

Trials will be eligible if they evaluated CBT or CBT+ interventions versus TAU or other psychosocial treatments, eligible treatments as defined in Treatment Comparisons Section.

Data Collection and Processes

IPD will be collected from principal investigators of past and on-going RCTs of CBT for psychosis in the UK and internationally. Our ability to successfully collect relevant IPD is facilitated by several factors. Our research team includes researchers who have conducted some of the largest RCTs in this area. Furthermore, we have established a network of collaborators to support the retrieval of relevant IPD: the CBTp IMPART Consortium. We will continue to expand the CBTp IMPART Consortium over the lifetime of the project by sending invitation emails to all researchers who have published and/or are currently conducting randomised controlled trials relevant to this work. Participating researchers will be sent specific data request forms outlining variables pertinent to the present IPD-MA. They will be asked to fully anonymise the requested dataset and share them with our research team using a safe data transfer system provided by the IT services University of Liverpool. We will remain in regular contact with all Consortium members throughout the lifetime of the project to clarify queries about their IPD and its integrity.

A statistical analysis plan detailing the data cleaning and coding, and the analyses to be conducted has been produced and will be available upon request. Data received will be

For peer review only

systematically recoded to ensure common scales or measurements across studies. We will liaise with principal investigators and statisticians of the primary studies to resolve any data issues and prepare the dataset for IPD-MA. In addition to primary and secondary outcomes data, IPD and relevant supporting material (e.g. trial codebook, therapy manuals, statistical analysis plans) will be requested to code the stated treatment groups, treatment effect modifiers, primary and secondary outcomes.

The primary outcome will be analysed on the PANSS scale, with comparable information recorded on other scales converted onto the PANSS scale where possible (e.g. Leucht et al [18] provide supplemental tables to convert between PANSS and BPRS). Secondary outcomes will be transformed onto the most commonly reported scale, where established conversion tables exist. If transformation to a common scale is impossible, or there is no most common scale, standardised values (calculated by dividing by the between patient variation) will be employed [19]. Transformation onto sub-scales will not be attempted. For example, secondary outcome Change in specific symptom clusters: Positive is defined as the sum of the positive sub-scale of the PANSS score. If a study records a comparable measure, no attempt will be made to transform this measure onto the positive sub-scale of PANSS, however the data would contribute to a standardized score analysis e.g. as part of a sensitivity analysis.

Statistical Analysis

Data Analysis

All randomised patients will be included, and an intention to treat (ITT) principle will be followed throughout. To examine IPD integrity and concordance with original trial analyses, all trials will be re-analysed individually and the original authors asked to confirm the individual study results and resolve any discrepancies. Throughout, a frequentist approach to analyses will be taken. All analyses will be conducted using R [20]. If the planned quantitative analyses cannot be undertaken, the data will be described qualitatively.We will examine the pooled treatment effect for each outcome by performing a series of one-step IPD meta-analyses (where IPD from individual studies are analysed simultaneously whilst

For peer review only

accounting for study-level clustering), and two-step IPD meta-analyses (where estimates of the treatment effect are initially computed from the IPD of each study, and then aggregated using conventional inverse variance meta-analytic approach) [6]. Throughout, due to anticipated heterogeneity between studies, a random effects approach will be employed (using a Dersimonian and Laird approach for two-stage analyses [21], and including a study level random treatment effect, and fixed study membership effect in one-stage approaches

Both one stage and two stage analyses will be conducted to allow a full investigation of the data [10]. The two stage analyses will be conducted initially, to help identify areas of higher between study heterogeneity through examination of e.g. forest plots of analyses. One stage analyses will then be conducted to allow multiple interactions between treatment modifiers to be examined. One and two stage analysis results will be compared, to confirm that areas of heterogeneity are identified similarly between each approach.

Due to considerable variation in the follow-up periods considered in the original trials, separate analysis of trials with highly comparable or identical points of assessment (e.g. 3 month, 6 month etc.) would be unfeasible. In order to maximise the data contributing to the analysis, outcomes measured at multiple time points will be modelled longitudinally. As dropout may be an issue during study periods, we will employ joint modelling methods (e.g. [22, 23]) to account for study dropout. Outcomes measured at a single time point (e.g. at the end of the treatment period) will be analysed using generalised linear models.

In two-stage analyses, treatment-modifier interactions will be estimated within each study, and the results pooled. In one-stage analyses, treatment-moderators interactions separating out within and between study effects will be examined, while accounting for clustering of participants within studies [6, 7, 24]. Any treatment effect modifier found to be significant at a level of 0.05 will be retained in a list of potential treatment effect modifiers. Treatment effect modifiers with a significant effect for each outcome will be identified through examination of 95% Confidence Intervals (CI). Confidence intervals for both joint and GLM analyses will be calculated through bootstrapping, using 200 bootstrap samples. Once this list has been compiled, both forward and backward manual selection procedures for model parameters will be conducted for the one and two stage analyses of the primary and secondary outcomes, provided sufficient data is available. The overlap between the

For peer review only

parameters selected by the forward and backward selection procedures will highlight, from the list of potential treatment effect modifiers, parameters most likely to be true treatment effect modifiers.

This investigation involves a large number of planned analyses. However, this analysis of treatment modifiers of CBT is, to an extent, exploratory. As such, this investigation aims to identify potential relationships, and in so doing motivate future investigations specifically targeting the identified areas of interest. Consequently, and given that there is not a standard multiple testing approach currently recommended for IPD-MA, we will not adjust for multiple testing, although we reiterated that these analyses are, to an extent, exploratory. If methods are developed during the course of this project that are recommended as standard to account for multiple testing in IPD-MA, application of the methods will be examined.

The main analyses will be conducted as complete case analyses, i.e. only those contributing data for all variables (outcome or explanatory) included in the model will be used in the meta-analyses. If the level of missing data for treatment effect modifiers or outcomes is large across the studies included in the meta-analysis, if possible, the effect of missing data on the conclusions of the analyses will be investigated by re-conducting the analyses of the primary and secondary outcomes based on multiply-imputed datasets, and the results compared to those obtained from the complete case analysis. This is in addition to the planned sensitivity analyses.

Heterogeneity, bias and study quality

This investigation employs a random effects approach to analyses. As such, in two-stage analyses, statistical heterogeneity will be examined using the τ^2 (which provides an estimate of between-study variance) and I^2 statistics (which provides the proportion of total variance that is due to "true" heterogeneity in treatment effects, interpretation as stated in the Cochrane Handbook [25]). Additionally, the p value for the χ^2 test for heterogeneity, along with visual inspection of forest plots, will be assessed. In one-stage analyses, heterogeneity can be assessed through the variance of the study level random treatment effect and through examination of the coefficients for fixed study membership terms.

For peer review only

If substantial heterogeneity is observed between results from different groups of studies, data across heterogeneous groups of studies will not be pooled, and the demographics and characteristics of the differing groups of studies will be compared in an attempt to identify differences that could cause the heterogeneity. Potential evidence of substantial heterogeneity in two stage analyses is defined in this investigation as some combination of (1) significant p value for χ^2test for heterogeneity in intervention tests at level of 0.10, (2) I^2 statistic greater than 50% (representing substantial heterogeneity), (3) visual inspection of the forest plot to identify heterogeneity (Cochrane Handbook, section 9.5.2 [26]). Potential evidence of substantial heterogeneity in one stage analyses will be given by comparison of models with and without the study level random treatment effect and fixed study membership terms. If evidence exists of heterogeneity not accounted for through the proposed model structure, use of additional terms (fixed effects or study level random effects) will be examined and noted.

Risk of bias in each study will be assed using the Cochrane Collaboration Risk of Bias tool . Analyses and results will be interpreted in light of the risk of bias of the studies included in the meta-analysis.

Publication bias (and other selection bias/small study effects) will be investigated through inspection of contour-enhanced funnel plots and appropriate statistical tests for funnel plot asymmetry [11-13]. Assessment of publication bias will only be undertaken for analyses containing 10 or more trials (due to the low power of the assessments for analyses containing small numbers of trials).

Analyses of the data will clearly report the proportion of individuals within each study for which IPD could be obtained, as well as the numbers of studies which were deemed eligible to be included in the meta-analysis, but which did not supply any data. Interpretation of overall strength of the evidence regarding modifiers of patient response to treatment examined in this evidence synthesis will be appraised in light of relevant assessment of IPD integrity, availability and risk of bias.

Subgroup and Sensitivity Analysis

Sensitivity analyses

For peer review only

For both primary and secondary outcomes, where conversion between scales has occurred, sensitivity analyses will be conducted that (1) remove studies with transformed data, analysing only data recorded on the main scale, and (2) use standardised scores across studies [19] (unless standardised scores have been used in the main analysis).

There were a range of ways in which some treatment effect modifiers could be coded in this investigation. As such, sensitivity analyses will also be conducted investigating whether the method of coding particular modifiers, effected the results. Sensitivity analyses will also be conducted to investigate unavailability of IPD (comparing results from two-stage MA that combine results from study specific IPD analyses, with AD extracted from study reports, to the main analysis based only on IPD), and to investigate changes in treatment effect over time (primary and longitudinally measured secondary outcome one and two-stage analyses will be re-conducted including an interaction term between treatment and time).

Subgroup Analyses

We will conduct subgroup analyses contrasting non-blind versus single-blind trials to examine the effect of masking of outcome assessments. Any analyses where outcomes have been transformed onto a common scale will also be sub-grouped as data originally recorded on the common scale versus data transformed onto the common scale. In one stage analyses, sub-grouping will be achieved by interacting the coefficient of interest with the grouping variable. In two stage analyses, results will be pooled from studies belonging to each sub-group.

Discussion

NICE and other clinical guidelines worldwide recommends CBT as an intervention for the treatment and management of psychosis and schizophrenia [2], however AD-MA to date have reported heterogeneous treatment effects between studies. This heterogeneity may be attributable to certain as yet unidentified patient or intervention specific characteristics that influence the clinical effectiveness of CBT. This IPD-MA examines a range of potential treatment effect modifiers for CBT for patients with patients with schizophrenia-spectrum diagnoses, for the primary outcome overall psychotic symptoms severity, as well as a range

For peer review only

of secondary outcomes commonly targeted by CBT for psychosis. This investigation is required to establish the effectiveness of CBT for psychosis across a range of different populations, and will provide evidence to improve healthcare for patients with schizophrenia-spectrum diagnoses.

Author Contributions

FV, CTS and APM conceived the idea for the review, and are project managing the systematic review and meta-analysis. GD provided input in the initial stages of the development and design of the project. EL provided patient and public involvement of the project. TK will be involved in future dissemination of research. CTS and MS drafted the Statistical Analysis Plan for the project with help from RE, and (along with XL) input into data collection, cleaning and analysis. All authors contributed to the development of the idea and drafting and revision of the manuscript. All authors gave approval for the manuscript to be submitted.

Funding

This project was funded by the NIHR Health Technology Assessment Programme (project number 15/187)

Competing interests

We recognise that one member of our team may be regarded as having a vested interest in CBT (Morrison is the only co-applicant actively involved in CBT training, leading of trial grants and receiving royalties from CBT texts or books). The other members of the team do not have conflicts of interest.

Patient consent

Not required as secondary analysis of data

For peer review only

Provenance and peer review

This project has been externally peer reviewed as part of the funding competition that supported this work. A protocol consistent with the information in this report has been sent to and approved by the HTA.

Author note

If there is need to amend this protocol, the date of each amendment and the reason for change will be noted. Each change will be scrutinised by the funder, with the date of approval recorded, and version number updated accordingly.

We used the PRISMA-P checklist when writing our report [27]

References

1. Stewart, L.A., et al., *Preferred reporting items for a systematic review and meta-analysis of individual participant data: The prisma-ipd statement.* JAMA, 2015. **313**(16): p. 1657-1665.
2. National Institute for Health and Care Excellence, *NICE guidelines CG178 - Psychosis and schizophrenia in adults: Treatment and management*. 2014, London: National Institute for Health and Care Excellence.
3. Wykes, T., et al., *Cognitive Behavior Therapy for Schizophrenia: Effect Sizes, Clinical Models, and Methodological Rigor.* Schizophrenia Bulletin, 2008. **34**(3): p. 523-537.

For peer review only

4. Jauhar, S., et al., *Cognitive-behavioural therapy for the symptoms of schizophrenia: systematic review and meta-analysis with examination of potential bias.* The British Journal of Psychiatry, 2014. **204**(1): p. 20-29.
5. Lambert, P.C., et al., *A comparison of summary patient-level covariates in meta-regression with individual patient data meta-analysis.* Journal of Clinical Epidemiology, 2002. **55**(1): p. 86-94.
6. Riley, R.D., P.C. Lambert, and G. Abo-Zaid, *Meta-analysis of individual participant data: rationale, conduct, and reporting.* BMJ, 2010. **340**.
7. Riley, R.D., et al., *Meta-analysis of continuous outcomes combining individual patient data and aggregate data.* Statistics in Medicine, 2008. **27**(11): p. 1870-1893.
8. Smith, C.T., et al., *Individual participant data meta-analyses compared with meta-analyses based on aggregate data.* Trials, 2011. **12**(Suppl 1): p. A57-A57.
9. Brabban, A., S. Tai, and D. Turkington, *Predictors of Outcome in Brief Cognitive Behavior Therapy for Schizophrenia.* Schizophrenia Bulletin, 2009. **35**(5): p. 859-864.
10. Rathod, S., et al., *Insight into schizophrenia: the effects of cognitive behavioural therapy on the components of insight and association with sociodemographics—data on*

For peer review only

a previously published randomised controlled trial. Schizophrenia Research, 2005. **74**(2–3): p. 211-219.

11. Allott, K., et al., *Patient predictors of symptom and functional outcome following cognitive behaviour therapy or befriending in first-episode psychosis.* Schizophrenia Research, 2011. **132**(2–3): p. 125-130.
12. Lincoln, T.M., et al., *Who stays, who benefits? Predicting dropout and change in cognitive behaviour therapy for psychosis.* Psychiatry Research, 2014. **216**(2): p. 198-205.
13. Hutton, P., et al., *Cognitive behavioural therapy for psychosis: A systematic review and meta-analysis* PROSPERO reference: CRD42013003911, 2013.
14. Hutton, P., et al., *Cognitive behavioural therapy for psychosis: rationale and protocol for a systematic review and meta-analysis.* Psychosis, 2014. **6**(3): p. 220-230.
15. Leucht, S., et al., *Dose equivalents for second-generation antipsychotics: The minimum effective dose method.* Schizophrenia Bulletin, 2014. **40**(2): p. 314-326.
16. Hermes, E.D.A., et al., *Minimum Clinically Important Difference in the Positive and Negative Syndrome Scale With Data From the Clinical Antipsychotic Trials of Intervention Effectiveness (CATIE).* JOURNAL OF CLINICAL PSYCHIATRY, 2012. **73**(4): p. 526-532.

For peer review only

17. Leucht, S., et al., *Linking the PANSS, BPRS, and CGI: clinical implications.* Neuropsychopharmacology: Official Publication Of The American College Of Neuropsychopharmacology, 2006. **31**(10): p. 2318-2325.

18. Leucht, S., et al., *Equipercentile linking of the BPRS and the PANSS.* European Neuropsychopharmacology, 2013. **23**(8): p. 956-959.

19. Higgins, J., *Meta-analysis of continuous outcome data from individual patients.* STATISTICS IN MEDICINE, 2001. **20**(15): p. 2219-2241.

20. R Core Team, *R: A Language and Environment for Statistical Computing*. 2015, R Foundation for Statistical Computing: Vienna, Austria.

21. DerSimonian, R. and N. Laird, *Meta-analysis in clinical trials.* Controlled Clinical Trials, 1986. **7**(3): p. 177-188.

22. Wulfsohn, M.S. and A.A. Tsiatis, *A Joint Model for Survival and Longitudinal Data Measured with Error.* International Biometric Society, 1997(1): p. 330.

23. Sudell, M., et al., *Investigation of 2-stage meta-analysis methods for joint longitudinal and time-to-event data through simulation and real data application.* Statistics in Medicine, 2017.

24. Hua, H., et al., *One-stage individual participant data meta-analysis models: estimation of treatment-covariate interactions must avoid ecological bias by separating out*

For peer review only

within-trial and across-trial information. Statistics in Medicine, 2017. **36**(5): p. 772-789.

25. Higgins, J., S. Green, and (editors). *Cochrane Handbook for Systematic Reviews of Interventions Version 5.1.0 (updated March 2011). The Cochrane Collaboration, 2011. Available from www.cochrane-handbook.org.*

26. *Deeks JJ, Higgins JPT, Altman DG (editors). Chapter 9: Analysing data and undertaking meta-analyses. In: Higgins JPT, Green S (editors). Cochrane Handbook for Systematic Reviews of Interventions Version 5.1.0 (updated March 2011). The Cochrane Collaboration, 2011. Available from www.cochrane-handbook.org.*

27. *Moher D, Shamseer L, Clarke M, Ghersi D, Liberati A, Petticrew M, Shekelle P, Stewart LA. Preferred Reporting Items for Systematic Review and Meta-Analysis Protocols (PRISMA-P) 2015 statement. Syst Rev. 2015;4(1):1..*

For peer review only

Reporting checklist for protocol of a systematic review.

Based on the PRISMA-P guidelines.

Instructions to authors

Complete this checklist by entering the page numbers from your manuscript where readers will find each of the items listed below.

Your article may not currently address all the items on the checklist. Please modify your text to include the missing information. If you are certain that an item does not apply, please write "n/a" and provide a short explanation.

Upload your completed checklist as an extra file when you submit to a journal.

In your methods section, say that you used the PRISMA-Preporting guidelines, and cite them as:

Moher D, Shamseer L, Clarke M, Ghersi D, Liberati A, Petticrew M, Shekelle P, Stewart LA. Preferred Reporting Items for Systematic Review and Meta-Analysis Protocols (PRISMA-P) 2015 statement. Syst Rev. 2015;4(1):1.

For peer review only

		Reporting Item	Page Number
**Title**			
Identification	#1a	Identify the report as a protocol of a systematic review	1
Update	#1b	If the protocol is for an update of a previous systematic review, identify as such	n/a
**Registration**			
	#2	If registered, provide the name of the registry (such as PROSPERO) and registration number	2
**Authors**			
Contact	#3a	Provide name, institutional affiliation, e-mail address of all protocol authors; provide physical mailing address of corresponding author	1
Contribution	#3b	Describe contributions of protocol authors and identify the guarantor of the review	15
**Amendments**			

	#4	If the protocol represents an amendment of a previously completed or published protocol, identify as such and list changes; otherwise, state plan for documenting important protocol amendments	n/a
**Support**			
Sources	#5a	Indicate sources of financial or other support for the review	16
Sponsor	#5b	Provide name for the review funder and / or sponsor	16
Role of sponsor or funder	#5c	Describe roles of funder(s), sponsor(s), and / or institution(s), if any, in developing the protocol	n/a
**Introduction**			
Rationale	#6	Describe the rationale for the review in the context of what is already known	2-3
Objectives	#7	Provide an explicit statement of the question(s) the review will address with reference to participants, interventions, comparators, and outcomes (PICO)	3-7
**Methods**			
Eligibility criteria	#8	Specify the study characteristics (such as PICO, study design, setting, time frame) and report characteristics (such as years considered, language, publication status) to be used as criteria for eligibility for the review	8-9
Information sources	#9	Describe all intended information sources (such as electronic databases, contact with study authors, trial registers or other grey literature sources) with planned dates of coverage	8
Search strategy	#10	Present draft of search strategy to be used for at least one electronic database, including planned limits, such that it could be repeated	8
Study records - data management	#11a	Describe the mechanism(s) that will be used to manage records and data throughout the review	10-11
Study records - selection process	#11b	State the process that will be used for selecting studies (such as two independent reviewers) through each phase of the review (that is, screening, eligibility and inclusion in meta-analysis)	8-9
Study records - data collection process	#11c	Describe planned method of extracting data from reports (such as piloting forms, done independently, in duplicate), any processes for	10-11

For peer review only

		obtaining and confirming data from investigators	
Data items	#12	List and define all variables for which data will be sought (such as PICO items, funding sources), any pre-planned data assumptions and simplifications	4-7
Outcomes and prioritization	#13	List and define all outcomes for which data will be sought, including prioritization of main and additional outcomes, with rationale	4-7
Risk of bias in individual studies	#14	Describe anticipated methods for assessing risk of bias of individual studies, including whether this will be done at the outcome or study level, or both; state how this information will be used in data synthesis	13-14
Data synthesis	#15a	Describe criteria under which study data will be quantitatively synthesised	11-15
Data synthesis	#15b	If data are appropriate for quantitative synthesis, describe planned summary measures, methods of handling data and methods of combining data from studies, including any planned exploration of consistency (such as I2, Kendall's τ)	11-15
Data synthesis	#15c	Describe any proposed additional analyses (such as sensitivity or subgroup analyses, meta-regression)	14-15
Data synthesis	#15d	If quantitative synthesis is not appropriate, describe the type of summary planned	11
Meta-bias(es)	#16	Specify any planned assessment of meta-bias(es) (such as publication bias across studies, selective reporting within studies)	13-14
Confidence in cumulative evidence	#17	Describe how the strength of the body of evidence will be assessed (such as GRADE)	14

The PRISMA-P checklist is distributed under the terms of the Creative Commons Attribution License CC-BY 4.0. This checklist was completed on 23. September 2019 using https://www.goodreports.org/, a tool made by the EQUATOR Network in collaboration with Penelope.ai

For peer review only

Protocol for Individual Participant Data Meta Analysis of randomised control trials of patients with psychosis, to investigate treatment effect modifiers for CBT versus treatment as usual or other psychosocial interventions

Journal:	*BMJ Open*
Manuscript ID	bmjopen-2019-035062.R1
Article Type:	Protocol
Date Submitted by the Author:	31-Jan-2020
Complete List of Authors:	Sudell, Maria; University of Liverpool, Department of Biostatistics, Institute of Translational Medicine<br>Tudur-Smith, Catrin; University of Liverpool, Biostatistics<br>Liao, Xiaomeng; University of Liverpool, Department of Biostatistics, Institute of Translational Medicine<br>Longden, Eleanor; Greater Manchester West Mental Health NHS Foundation Trust<br>Dunn, Graham; University of Manchester, Health Methodology Research<br>Kendall, Tim; National Collaborating Centre for Mental Health<br>Emsley, Richard; King’s College London, Department of Biostatistics and Health Informatics, Institute of Psychiatry, Psychology and Neuroscience<br>Morrison, Anthony; University of Manchester<br>Varese, Filippo; Manchester Academic Health Science Centre,
**Primary Subject Heading**:	Mental health
Secondary Subject Heading:	Research methods
Keywords:	IPD-MA, Treatment Modifier, CBT, Psychosis

SCHOLARONE™ Manuscripts

I, the Submitting Author has the right to grant and does grant on behalf of all authors of the Work (as defined in the below author licence), an exclusive licence and/or a non-exclusive licence for contributions from authors who are: i) UK Crown employees; ii) where BMJ has agreed a CC-BY licence shall apply, and/or iii) in accordance with the terms applicable for US Federal Government officers or employees acting as part of their official duties; on a worldwide, perpetual, irrevocable, royalty-free basis to BMJ Publishing Group Ltd ("BMJ") its licensees and where the relevant Journal is co-owned by BMJ to the co-owners of the Journal, to publish the Work in this journal and any other BMJ products and to exploit all rights, as set out in our licence.

The Submitting Author accepts and understands that any supply made under these terms is made by BMJ to the Submitting Author unless you are acting as an employee on behalf of your employer or a postgraduate student of an affiliated institution which is paying any applicable article publishing charge ("APC") for Open Access articles. Where the Submitting Author wishes to make the Work available on an Open Access basis (and intends to pay the relevant APC), the terms of reuse of such Open Access shall be governed by a Creative Commons licence – details of these licences and which Creative Commons licence will apply to this Work are set out in our licence referred to above.

Other than as permitted in any relevant BMJ Author's Self Archiving Policies, I confirm this Work has not been accepted for publication elsewhere, is not being considered for publication elsewhere and does not duplicate material already published. I confirm all authors consent to publication of this Work and authorise the granting of this licence.

For peer review only

Protocol for Individual Participant Data Meta Analysis of randomised control trials of patients with psychosis, to investigate treatment effect modifiers for CBT versus treatment as usual or other psychosocial interventions

Maria Sudell[1,a*], Catrin Tudur Smith[1,b], Xiaomeng Liao[1,c], Eleanor Longden[3,d], Graham Dunn[4], Tim Kendall[5, 6,e], Richard Emsley[7,f] & Anthony P. Morrison[2,3,g] Filippo Varese[2,3,h]

[1]Institute of Translational Medicine, University of Liverpool; [2]School of Health Sciences, Division of Psychology and Mental Health, Faculty of Biology Medicine and Health, University of Manchester; [3]Greater Manchester Mental Health NHS Mental Health Foundation Trust; [4]Institute of Population Health, University of Manchester; [5]National Collaborating Centre for Mental Health; [6]Sheffield Health and Social Care NHS Foundation Trust; [7]Department of Biostatistics & Health Informatics, Kings College London; [a]mesudell@liverpool.ac.uk; [b]cat1@liverpool.ac.uk; [c]Xiaomeng.Liao@liverpool.ac.uk; [d]eleanorlongden@googlemail.com; [e]tim2.kendall@virgin.net; [f]richard.emsley@kcl.ac.uk; [g]Anthony.P.Morrison@manchester.ac.uk ; [h]filippo.varese@manchester.ac.uk;

**Corresponding author*

University of Manchester
Division of Psychology and Mental Health
School of Health Sciences
Zochonis Building, 2nd floor; room 2.40
Manchester
M13 9PL

Article Summary

Introduction: Aggregate data meta-analyses have shown heterogeneous treatment effects for Cognitive Behavioural Therapy (CBT) for patients with schizophrenia-spectrum

diagnoses. This heterogeneity could stem from specific intervention or patient characteristics that could influence the clinical effectiveness of CBT, termed treatment effect modifiers. This Individual Participant Data Meta-Analysis (IPD-MA) will investigate a range of potential treatment effect modifiers of the efficacy CBT. **Methods and analysis**: We will perform a systematic review and meta-analysis of studies investigating CBT vs treatment as usual (TAU), or CBT vs other psychosocial interventions, for patients with schizophrenia-spectrum diagnoses. The Cochrane Central Register of Controlled Trials (CENTRAL), PubMed, EMBASE and the online clinical trials registers of the US government, European Union, World Health Organization and Current Controlled Trials Ltd will be searched. Two researchers will screen titles and abstracts identified by the search. Individual participant data will be requested for any eligible study, for the primary outcome (overall psychotic symptoms), secondary outcomes and treatment effect modifiers. Data will be checked and recoded according to an established Statistical Analysis Plan. One and two-stage random effects meta-analyses investigating potential treatment effect modifiers will be conducted. A list of potential treatment effect modifiers for CBT will be produced, motivating future research into particular modifiers. **Ethics and dissemination:** This study does not require ethical approval as it is based on data from existing studies, although best ethical practice for secondary analysis of clinical data will be followed. The findings will be submitted for publication in peer-reviewed journals, and promoted to relevant stakeholders.

Prospero registration number CRD42017060068

Strengths and Limitations of study

- This will be the first published IPD-MA to investigate treatment effect modifiers for CBT for patients with schizophrenia-spectrum diagnoses
- The review will consider the efficacy of CBT across multiple outcomes of interest in addition to psychotic symptoms severity
- The search will be conducted without geographical, language or time restrictions
- A potential limitation of this study will be the degree of heterogeneity between studies in recorded measures and scales employed.

For peer review only

Introduction

Cognitive behavioural therapy (CBT) is a recommended intervention for the treatment and management of psychosis and schizophrenia [1]. Aggregate Data Meta-Analyses (AD-MA) suggest that CBT for psychosis has modest but considerably heterogeneous treatment effects (for example [2, 3]). This inconsistency partly stems from inter-trial variation in several key methodological characteristics of the existing Randomised Controlled Trials (RCT; for example, blinding/masking of outcome assessments [2, 3]) but could also reflect the impact of unaccounted clinical heterogeneity i.e. specific intervention and patient characteristics that can potentially influence the clinical effectiveness of CBT. For instance, previous trials differed widely in terms of intervention characteristics (e.g. number of treatment sessions, treatment duration, use of manualised interventions), patients' baseline severity of psychotic and other comorbid symptoms, their demographic characteristics (e.g. age, gender and ethnic origin), and illness duration. The identification of moderators of treatment response and/or sub-groups of patients who may particularly benefit from CBT would allow optimisation of treatment delivery, with significant implications in terms of improved clinical effectiveness, cost savings and maximisation of patients' informed choice of treatment.

The impact of these potential treatment effect modifiers, however, remains unaccounted for (or at best poorly estimated) in AD-MA due to their reliance of the reporting quality of primary studies and the limited statistical power of "standard" meta-analytic methods for testing treatment effect moderators [4]. Additional primary research would be costly and impractical given the large sample size required. The only approach suited for this type of research is Individual Patient Data Meta-Analysis (IPD-MA), a research synthesis method which summarises the evidence on a particular clinical question by considering individual participant level rather than aggregate level data from multiple related studies. IPD-MA allow (1) greater ability to examine the impact of multiple individual-level and study-level factors (and their combination) on the treatment effects considered, (2) standardisation of statistical methods used across studies, and (3) the potential of reduced risk of bias e.g. due

to selective reporting of outcomes compared to conventional AD-MA [5-8]. This article is the protocol for an IPD-MA to assess the impact of treatment effect modifiers of CBT.

Methods

Objectives of IPD-MA

Primary outcome

The primary objective of the IPD-MA of RCTs is to identify treatment effect modifiers for CBT or CBT+ vs Treatment As Usual (TAU) or other psychosocial interventions on overall psychotic symptoms severity as measured by PANSS scores [9, 10] in patients with schizophrenia-spectrum diagnoses.

Secondary outcomes

In this IPD-MA of RCTs, treatment effect modifiers for CBT or CBT+ vs TAU or other psychosocial interventions will also be examined for patients with schizophrenia-spectrum diagnoses for the secondary outcomes listed in Table 1**Error! Reference source not found.**.

For some secondary outcomes, multiple sub-outcomes will be examined, such as Change in severity of affective symptoms, which is examined both for anxiety and depression. Adverse event information will be sought specifically for the 4 sub-outcomes mentioned, but additional adverse event information will also be tabulated. Secondary outcomes were selected based on (1) outcomes often targeted in CBT for psychosis, (2) measures identified as valuable in our PPI consultations.

**Minimum clinical important differences (MCIDs) in PANSS scores [9-12]**
Reduction of ≥ 11 points
Reduction of ≥ 15 points
**Clinically Significant Deterioration (CSDs) in PANSS scores [9-12]**
Increase of ≥ 11 points
Increase of ≥ 15 points

**Change in specific symptom clusters**	
	Positive psychopathology
	Negative psychopathology
	General psychopathology
**Change in specific symptoms often targeted in CBT for psychosis**	
	Hallucinations severity
	Delusions severity
	Hallucinations associated subjective distress
	Delusions associated subjective distress
	Paranoia severity
**Change in severity of affective symptoms**	
	Anxiety
	Depression
**Subjectively defined recovery**	
**Quality of Life**	
**Social and Occupational functioning**	
**Early Treatment Discontinuation**	
**Adverse effects**	
	Deaths
	Attempts at suicide
	Suicide ideation
	Serious violent incidents
**Hospital readmissions**	

Table 1: Secondary outcomes

Treatment comparisons

Consistent with the analytic approaches in recent AD-MA [13, 14] our IPD-MA will distinguish between "pure" CBT interventions as defined by NICE [1] and "CBT+" interventions, where CBT+ is defined as a CBT treatment packages incorporating significant elements of other, distinct psychosocial intervention approaches (e.g. mindfulness, motivational interviewing, family intervention) alongside core CBT elements. CBT and CBT+

DRAFT
For peer review only

will be compared to Treatment As Usual (TAU) or other psychosocial interventions in individuals with a diagnosis of schizophrenia-spectrum disorders. TAU is defined as the level of care service users would routinely receive had they not been involved in the trial, other psychosocial interventions is defined as TAU supplemented by additional psychological or social interventions e.g. family therapy. Although all trials of CBT against these comparators will be eligible, these will be synthetized and contrasted in separate analyses for: (1) CBT vs TAU, (2) CBT vs other psychosocial interventions, (3) CBT+ vs TAU, (4) CBT+ vs other psychosocial interventions.

Treatment Effect Modifiers

The selection of treatment effect modifiers was informed by (1) knowledge of variables examined in previous and on-going RCTs by members of our team and the collaborators in the CBTp: IMPART Consortium; (2) the findings of studies which examined predictors of outcomes in previous RCTs [e.g. 11, 15-17], and (3) consultation meetings with service-users with psychosis and clinical psychologists and CBT therapists working with clients with psychosis in secondary care settings in the UK.

The treatment effect modifiers shown in Table 2**Error! Reference source not found.** will be investigated for both the primary and secondary outcomes. Treatment effect modifiers have been separated into three main groups: (1) Participant Demographic Characteristics, (2) Participant's Clinical (3) Specific Intervention Characteristics

	Treatment effect Modifier
**Participant's Demographic Characteristics**	Age at entry to trial
	Gender
	Ethnicity
**Participant's Clinical Characteristics**	Effect of specific diagnostic sub-groups

DRAFT For peer review only

	Phase of illness (first episode psychosis / multiple episode psychosis)
	Illness Duration
	Duration of untreated psychosis
	Initial Severity of Psychotic Symptoms (measured by baseline PANSS scores)
	Initial severity of comorbid affective symptoms (measured by baseline Anxiety scores)
	Initial severity of comorbid affective symptoms (measured by baseline Depression scores)
	Dosage equivalence of baseline anti-psychotic medication(s) [12]
	Number of anti-psychotic medications received at baseline)
**Specific Intervention Characteristics**	time period over which treatment was delivered*
	number of therapy sessions offered in the study*
	number of therapy sessions attended by the individual
	Minimum study required level of therapist's training and competence*;

DRAFT
For peer review only

	Measures of therapeutic alliance;
	Use of manualised interventions*;
	Use of formulation-based interventions*;
	Indicator for whether the intervention was designed to target the outcome under scrutiny*;
	Individual versus group interventions*
	Length of study follow up

*Table 2: Treatment effect modifiers examined in this IPD-MA. Note: treatment effect modifiers marked with * are study level variables, the remaining are individual level variables*

Protocol

This evidence synthesis will follow state-of-the-art guidelines for IPD-MA, and our outputs will comply as a minimum with the PRISMA statement for the reporting of IPD-MA [8]. The search strategy of our IPD-MA builds on the protocol and database searches carried out as part of a recent AD-MA carried out by members of our team [13, 14]. Our study selection criteria are consistent with those employed in this recent AD-MA. Similarly, our literature searches will update those carried out as part of this review to identify any RCTs that have become available since the date of search.

Search Strategy

We will update the searches already conducted as part of a recent AD-MA [13, 14] to identify any trials that might have been become available for research synthesis since the date of last search. Database searches will be conducted on the Cochrane Central Register of Controlled Trials (CENTRAL), PubMed, EMBASE and the online clinical trials registers of

the US government, European Union, World Health Organization and Current Controlled Trials Ltd.

In line with the protocol of the AD-MA carried out by members of our team [13, 14], titles, abstracts and keywords will be searched in the publication databases using the adaptations of following generic strategy: (schizo$ [exp. schizophrenia + psychosis + schizoaffective]) AND (trial [exp. RCT +controlled trial + clinical trial]) AND (cbt [exp. cognitive therapy + behaviour therapy +psychotherapy]).

Selection of Studies

The project's PI and another member of the research team will screen titles and abstracts for relevance, and subsequently assess eligibility by examining the full-text reports against the above-mentioned criteria. When required, additional information to ascertain eligibility will be requested from the RCTs' authors and through the examination of treatment manual when available (e.g. to ascertain that interventions complied with NICE operational definitions of CBT). Discrepancies in selection decisions will be discussed, and arbitration by other members of the research team sought to achieve consensus. We will include studies based on the following eligibility criteria:

Participants

Trials where > 50% of participants have diagnoses in the schizophrenia-spectrum (schizophrenia, schizoaffective disorder or early psychosis) will be eligible. Trials where > 50% participants have an established diagnosis of bipolar disorder, intellectual disability, psychosis secondary to a general medical condition or organic pathology, or a primary diagnosis of substance-induced psychosis will be excluded. No restriction will be placed on participants' age, ethnicity, illness severity and illness duration.

For peer review only
DRAFT

Treatment Comparisons

Included studies must compare treatments that fit into one or more of the four stated treatment categories (CBT, CBT+, TAU, other psychosocial interventions). Study interventions will be classified into one of these four categories by clinicians involved with this project.

Outcomes

The study must provide data for one or more of the stated primary or secondary outcomes. Data can be recorded on any comparable scale, at any time-point. Data will also be sought for the treatment effect modifiers listed in Table 2**Error! Reference source not found.**.

Study Designs

Parallel single-blind or open controlled trials with at least two arms utilizing random allocation to treatment will be considered for inclusion. Single arm or cross-over studies will not be eligible for inclusion in the review. Studies employing other research designs (case series, cohort analyses) will not be eligible.

Trials will be eligible if they evaluated CBT or CBT+ interventions versus TAU or other psychosocial treatments, eligible treatments as defined in Treatment Comparisons Section.

Data Collection and Processes

IPD will be collected from principal investigators of past and on-going RCTs of CBT for psychosis in the UK and internationally. Our ability to successfully collect relevant IPD is facilitated by several factors. Our research team includes researchers who have conducted some of the largest RCTs in this area. Furthermore, we have established a network of collaborators to support the retrieval of relevant IPD: the CBTp IMPART Consortium. We will continue to expand the CBTp IMPART Consortium over the lifetime of the project by sending invitation emails to all researchers who have published and/or are currently conducting randomised controlled trials relevant to this work. Participating researchers will be sent specific data request forms outlining variables pertinent to the present IPD-MA. They will be

DRAFT
For peer review only

asked to fully anonymise the requested dataset and share them with our research team using a safe data transfer system provided by the IT services University of Liverpool. We will remain in regular contact with all Consortium members throughout the lifetime of the project to clarify queries about their IPD and its integrity.

In the case of no response to data requests (defined as a minimum of 4 contact attempts with no response), details of the study would be stated as "non-acquired data". Details concerning the number and proportion of studies and individuals for which IPD has been obtained will be stated in reports of analyses. Additionally, we have planned sensitivity analyses to assess the impact of non-acquired data on results of the project, which will combine obtained IPD, with AD from studies not supplying IPD.

A statistical analysis plan detailing the data cleaning and coding, and the analyses to be conducted has been produced and will be available upon request. Data received will be systematically recoded to ensure common scales or measurements across studies. We will liaise with principal investigators and statisticians of the primary studies to resolve any data issues and prepare the dataset for IPD-MA. In addition to primary and secondary outcomes data, IPD and relevant supporting material (e.g. trial codebook, therapy manuals, statistical analysis plans) will be requested to code the stated treatment groups, treatment effect modifiers, primary and secondary outcomes.

The primary outcome will be analysed on the PANSS scale, with comparable information recorded on other scales converted onto the PANSS scale where possible (e.g. Leucht et al [18] provide supplemental tables to convert between PANSS and BPRS). Secondary outcomes will be transformed onto the most commonly reported scale, where established conversion tables exist. If transformation to a common scale is impossible, or there is no most common scale, standardised values (calculated by dividing by the between patient variation) will be employed [19]. Transformation onto sub-scales will not be attempted. For example, secondary outcome Change in specific symptom clusters: Positive is defined as the sum of the positive sub-scale of the PANSS score. If a study records a comparable measure, no attempt will be made to transform this measure onto the positive sub-scale of PANSS, however the data would contribute to a standardized score analysis e.g. as part of a sensitivity analysis.

For peer review only - http://bmjopen.bmj.com/site/about/guidelines.xhtml

Statistical Analysis

Data Analysis

All randomised patients will be included, and an intention to treat (ITT) principle will be followed throughout. To examine IPD integrity and concordance with original trial analyses, all trials will be re-analysed individually and the original authors asked to confirm the individual study results and resolve any discrepancies. Throughout, a frequentist approach to analyses will be taken. All analyses will be conducted using R [20]. If the planned quantitative analyses cannot be undertaken, the data will be described qualitatively.We will examine the pooled treatment effect for each outcome by performing a series of one-step IPD meta-analyses (where IPD from individual studies are analysed simultaneously whilst accounting for study-level clustering), and two-step IPD meta-analyses (where estimates of the treatment effect are initially computed from the IPD of each study, and then aggregated using conventional inverse variance meta-analytic approach) [5]. Throughout, due to anticipated heterogeneity between studies, a random effects approach will be employed (using a Dersimonian and Laird approach for two-stage analyses [21], and including a study level random treatment effect, and fixed study membership effect in one-stage approaches

Both one stage and two stage analyses will be conducted to allow a full investigation of the data [22]. The two stage analyses will be conducted initially, to help identify areas of higher between study heterogeneity through examination of e.g. forest plots of analyses. One stage analyses will then be conducted to allow multiple interactions between treatment modifiers to be examined. One and two stage analysis results will be compared, to confirm that areas of heterogeneity are identified similarly between each approach.

Due to considerable variation in the follow-up periods considered in the original trials, separate analysis of trials with highly comparable or identical points of assessment (e.g. 3 month, 6 month etc.) would be unfeasible. In order to maximise the data contributing to the analysis, outcomes measured at multiple time points will be modelled longitudinally. As dropout may be an issue during study periods, we will employ joint modelling methods (e.g. [23, 24]) to account for study dropout. Outcomes measured at a single time point (e.g. at the end of the treatment period) will be analysed using generalised linear models.

DRAFT For peer review only

In two-stage analyses, treatment-modifier interactions will be estimated within each study, and the results pooled. In one-stage analyses, treatment-moderators interactions separating out within and between study effects will be examined, while accounting for clustering of participants within studies [5, 6, 25]. Any treatment effect modifier found to be significant at a level of 0.05 will be retained in a list of potential treatment effect modifiers. Treatment effect modifiers with a significant effect for each outcome will be identified through examination of 95% Confidence Intervals (CI). Confidence intervals for both joint and GLM analyses will be calculated through bootstrapping, using 200 bootstrap samples. Once this list has been compiled, both forward and backward manual selection procedures for model parameters will be conducted for the one and two stage analyses of the primary and secondary outcomes, provided sufficient data is available. The overlap between the parameters selected by the forward and backward selection procedures will highlight, from the list of potential treatment effect modifiers, parameters most likely to be true treatment effect modifiers.

This investigation involves a large number of planned analyses. However, this analysis of treatment modifiers of CBT is, to an extent, exploratory. As such, this investigation aims to identify potential relationships, and in so doing motivate future investigations specifically targeting the identified areas of interest. Consequently, and given that there is not a standard multiple testing approach currently recommended for IPD-MA, we will not adjust for multiple testing, although we reiterated that these analyses are, to an extent, exploratory. If methods are developed during the course of this project that are recommended as standard to account for multiple testing in IPD-MA, application of the methods will be examined.

The main analyses will be conducted as complete case analyses, i.e. only those contributing data for all variables (outcome or explanatory) included in the model will be used in the meta-analyses. If the level of missing data for treatment effect modifiers or outcomes is large across the studies included in the meta-analysis, if possible, the effect of missing data on the conclusions of the analyses will be investigated by re-conducting the analyses of the primary and secondary outcomes based on multiply-imputed datasets, and the results compared to those obtained from the complete case analysis. This is in addition to the planned sensitivity analyses.

Heterogeneity, bias and study quality

This investigation employs a random effects approach to analyses. As such, in two-stage analyses, statistical heterogeneity will be examined using the τ^2 (which provides an estimate of between-study variance) and I^2 statistics (which provides the proportion of total variance that is due to "true" heterogeneity in treatment effects, interpretation as stated in the Cochrane Handbook [26]). Additionally, the p value for the χ^2 test for heterogeneity, along with visual inspection of forest plots, will be assessed. In one-stage analyses, heterogeneity can be assessed through the variance of the study level random treatment effect and through examination of the coefficients for fixed study membership terms.

If substantial heterogeneity is observed between results from different groups of studies, data across heterogeneous groups of studies will not be pooled, and the demographics and characteristics of the differing groups of studies will be compared in an attempt to identify differences that could cause the heterogeneity. Potential evidence of substantial heterogeneity in two stage analyses is defined in this investigation as some combination of (1) significant p value for χ^2test for heterogeneity in intervention tests at level of 0.10, (2) I^2 statistic greater than 50% (representing substantial heterogeneity), (3) visual inspection of the forest plot to identify heterogeneity (Cochrane Handbook, section 9.5.2 [27]). Potential evidence of substantial heterogeneity in one stage analyses will be given by comparison of models with and without the study level random treatment effect and fixed study membership terms. If evidence exists of heterogeneity not accounted for through the proposed model structure, use of additional terms (fixed effects or study level random effects) will be examined and noted.

Risk of bias in each study will be assed using the Cochrane Collaboration Risk of Bias tool . Analyses and results will be interpreted in light of the risk of bias of the studies included in the meta-analysis.

Publication bias (and other selection bias/small study effects) will be investigated through inspection of contour-enhanced funnel plots and appropriate statistical tests for funnel plot asymmetry [11,13,17]. Assessment of publication bias will only be undertaken for analyses

containing 10 or more trials (due to the low power of the assessments for analyses containing small numbers of trials).

Analyses of the data will clearly report the proportion of individuals within each study for which IPD could be obtained, as well as the numbers of studies which were deemed eligible to be included in the meta-analysis, but which did not supply any data. Interpretation of overall strength of the evidence regarding modifiers of patient response to treatment examined in this evidence synthesis will be appraised in light of relevant assessment of IPD integrity, availability and risk of bias.

Subgroup and Sensitivity Analysis

Sensitivity analyses

For both primary and secondary outcomes, where conversion between scales has occurred, sensitivity analyses will be conducted that (1) remove studies with transformed data, analysing only data recorded on the main scale, and (2) use standardised scores across studies [19] (unless standardised scores have been used in the main analysis).

There were a range of ways in which some treatment effect modifiers could be coded in this investigation. As such, sensitivity analyses will also be conducted investigating whether the method of coding particular modifiers, effected the results. Sensitivity analyses will also be conducted to investigate unavailability of IPD (comparing results from two-stage MA that combine results from study specific IPD analyses, with AD extracted from study reports, to the main analysis based only on IPD), and to investigate changes in treatment effect over time (primary and longitudinally measured secondary outcome one and two-stage analyses will be re-conducted including an interaction term between treatment and time).

Subgroup Analyses

We will conduct subgroup analyses contrasting non-blind versus single-blind trials to examine the effect of masking of outcome assessments. Any analyses where outcomes have been transformed onto a common scale will also be sub-grouped as data originally recorded on the common scale versus data transformed onto the common scale. In one stage analyses, sub-grouping will be achieved by interacting the coefficient of interest with the

For peer review only - http://bmjopen.bmj.com/site/about/guidelines.xhtml

grouping variable. In two stage analyses, results will be pooled from studies belonging to each sub-group.

Patient and Public Involvement

Throughout this project, we have taken care to involve key stakeholders in the design of the research. The research team contains a PPI representative. Additionally, secondary outcomes and treatment effect modifiers were identified in part through consultation meetings with service-users with psychosis and clinical psychologists and CBT therapists working with clients with psychosis in secondary care settings in the UK.

Results of this research will be disseminated to stakeholders via a range of methods including the preparation of an information pack for service users and carers, which will be made freely available online.

Ethics and Dissemination

This IPD-MA will be conducted in line with current recommendations of secondary analysis of IPD data (e.g. [5]). Specific ethics approval for the IPD-MA is not required, as the objectives of the IPD-MA are consistent with those of the original trials, and do not violate the condition of consent under which the data was collected. Throughout, anonymised data will be sought from study authors. Information from data owners should not include any personally identifiable information. Data and accompanying documentation will be held on a secure server by the research team. Findings of this research will be submitted for publication in high impact peer-reviewed journals, promoted to relevant stakeholders and presented to relevant research communities at international conferences.

Discussion

NICE and other clinical guidelines worldwide recommends CBT as an intervention for the treatment and management of psychosis and schizophrenia [1], however AD-MA to date have reported heterogeneous treatment effects between studies. This heterogeneity may be attributable to certain as yet unidentified patient or intervention specific characteristics

For peer review only - http://bmjopen.bmj.com/site/about/guidelines.xhtml

that influence the clinical effectiveness of CBT. This IPD-MA examines a range of potential treatment effect modifiers for CBT for patients with patients with schizophrenia-spectrum diagnoses, for the primary outcome overall psychotic symptoms severity, as well as a range of secondary outcomes commonly targeted by CBT for psychosis. The treatment effect modifiers and outcomes investigated in this project are wide ranging, however given the lack of current research in this area, it is hoped that this exploratory IPD-MA will provide guidance for future focussed research. This investigation is required to establish the effectiveness of CBT for psychosis across a range of different populations, and will provide evidence to improve healthcare for patients with schizophrenia-spectrum diagnoses.

Author Contributions

FV, CTS and APM conceived the idea for the review, and are project managing the systematic review and meta-analysis. GD provided input in the initial stages of the development and design of the project. EL provided patient and public involvement of the project. TK will be involved in future dissemination of research. CTS and MS drafted the Statistical Analysis Plan for the project with help from RE, and (along with XL) input into data collection, cleaning and analysis. All authors contributed to the development of the idea and drafting and revision of the manuscript. All authors gave approval for the manuscript to be submitted.

Funding

This project is funded by the National Institute for Health Research (NIHR) Health Technology Assessment Programme (project number 15/187/05). The views expressed are those of the author(s) and not necessarily those of the NIHR or the Department of Health and Social Care.

Acknowledgements

We thank all those involved in the PPI consultations that helped to shape this research project.

Competing interests

We recognise that one member of our team may be regarded as having a vested interest in CBT (Morrison is the only co-applicant actively involved in CBT training, leading of trial grants and receiving royalties from CBT texts or books). The other members of the team do not have conflicts of interest.

Patient consent

Not required as secondary analysis of data

Provenance and peer review

This project has been externally peer reviewed as part of the funding competition that supported this work. A protocol consistent with the information in this report has been sent to and approved by the HTA.

Author note

If there is need to amend this protocol, the date of each amendment and the reason for change will be noted. Each change will be scrutinised by the funder, with the date of approval recorded, and version number updated accordingly.
We used the PRISMA-P checklist when writing our report

References

1. National Institute for Health and Care Excellence, *NICE guidelines CG178 - Psychosis and schizophrenia in adults: Treatment and management*. 2014, London: National Institute for Health and Care Excellence.

2. Wykes, T., et al., *Cognitive Behavior Therapy for Schizophrenia: Effect Sizes, Clinical Models, and Methodological Rigor.* Schizophrenia Bulletin, 2008. **34**(3): p. 523-537.
3. Jauhar, S., et al., *Cognitive-behavioural therapy for the symptoms of schizophrenia: systematic review and meta-analysis with examination of potential bias.* The British Journal of Psychiatry, 2014. **204**(1): p. 20-29.
4. Lambert, P.C., et al., *A comparison of summary patient-level covariates in meta-regression with individual patient data meta-analysis.* Journal of Clinical Epidemiology, 2002. **55**(1): p. 86-94.
5. Riley, R.D., P.C. Lambert, and G. Abo-Zaid, *Meta-analysis of individual participant data: rationale, conduct, and reporting.* BMJ, 2010. **340**.
6. Riley, R.D., et al., *Meta-analysis of continuous outcomes combining individual patient data and aggregate data.* Statistics in Medicine, 2008. **27**(11): p. 1870-1893.
7. Smith, C.T., et al., *Individual participant data meta-analyses compared with meta-analyses based on aggregate data.* Trials, 2011. **12**(Suppl 1): p. A57-A57.
8. Stewart, L.A., et al., *Preferred reporting items for a systematic review and meta-analysis of individual participant data: The prisma-ipd statement.* JAMA, 2015. **313**(16): p. 1657-1665.

DRAFT

For peer review only

9. Hermes, E.D.A., et al., *Minimum Clinically Important Difference in the Positive and Negative Syndrome Scale With Data From the Clinical Antipsychotic Trials of Intervention Effectiveness (CATIE).* JOURNAL OF CLINICAL PSYCHIATRY, 2012. **73**(4): p. 526-532.
10. Leucht, S., et al., *Linking the PANSS, BPRS, and CGI: clinical implications.* Neuropsychopharmacology: Official Publication Of The American College Of Neuropsychopharmacology, 2006. **31**(10): p. 2318-2325.
11. Lincoln, T.M., et al., *Who stays, who benefits? Predicting dropout and change in cognitive behaviour therapy for psychosis.* Psychiatry Research, 2014. **216**(2): p. 198-205.
12. Leucht, S., et al., *Dose equivalents for second-generation antipsychotics: The minimum effective dose method.* Schizophrenia Bulletin, 2014. **40**(2): p. 314-326.
13. Hutton, P., et al., *Cognitive behavioural therapy for psychosis: A systematic review and meta-analysis* PROSPERO reference: CRD42013003911, 2013.
14. Hutton, P., et al., *Cognitive behavioural therapy for psychosis: rationale and protocol for a systematic review and meta-analysis.* Psychosis, 2014. **6**(3): p. 220-230.
15. Brabban, A., S. Tai, and D. Turkington, *Predictors of Outcome in Brief Cognitive Behavior Therapy for*

Schizophrenia. Schizophrenia Bulletin, 2009. **35**(5): p. 859-864.

16. Rathod, S., et al., *Insight into schizophrenia: the effects of cognitive behavioural therapy on the components of insight and association with sociodemographics—data on a previously published randomised controlled trial.* Schizophrenia Research, 2005. **74**(2–3): p. 211-219.
17. Allott, K., et al., *Patient predictors of symptom and functional outcome following cognitive behaviour therapy or befriending in first-episode psychosis.* Schizophrenia Research, 2011. **132**(2–3): p. 125-130.
18. Leucht, S., et al., *Equipercentile linking of the BPRS and the PANSS.* European Neuropsychopharmacology, 2013. **23**(8): p. 956-959.
19. Higgins, J., *Meta-analysis of continuous outcome data from individual patients.* STATISTICS IN MEDICINE, 2001. **20**(15): p. 2219-2241.
20. R Core Team, *R: A Language and Environment for Statistical Computing*. 2015, R Foundation for Statistical Computing: Vienna, Austria.
21. DerSimonian, R. and N. Laird, *Meta-analysis in clinical trials.* Controlled Clinical Trials, 1986. **7**(3): p. 177-188.
22. Burke, D.L., J. Ensor, and R.D. Riley, *Meta-analysis using individual participant data: one-stage and two-stage*

approaches, and why they may differ. Statistics in Medicine, 2017. **36**(5): p. 855-875.

23. Wulfsohn, M.S. and A.A. Tsiatis, *A Joint Model for Survival and Longitudinal Data Measured with Error.* International Biometric Society, 1997(1): p. 330.
24. Sudell, M., et al., *Investigation of 2-stage meta-analysis methods for joint longitudinal and time-to-event data through simulation and real data application.* Statistics in Medicine, 2017.
25. Hua, H., et al., *One-stage individual participant data meta-analysis models: estimation of treatment-covariate interactions must avoid ecological bias by separating out within-trial and across-trial information.* Statistics in Medicine, 2017. **36**(5): p. 772-789.
26. Higgins, J., S. Green, and (editors). *Cochrane Handbook for Systematic Reviews of Interventions Version 5.1.0 (updated March 2011). The Cochrane Collaboration, 2011. Available from www.cochrane-handbook.org.*
27. *Deeks JJ, Higgins JPT, Altman DG (editors). Chapter 9: Analysing data and undertaking meta-analyses. In: Higgins JPT, Green S (editors). Cochrane Handbook for Systematic Reviews of Interventions Version 5.1.0 (updated March 2011). The Cochrane Collaboration, 2011. Available from www.cochrane-handbook.org.*

For peer review only

DRAFT
For peer review only

Reporting checklist for protocol of a systematic review.

Based on the PRISMA-P guidelines.

Instructions to authors

Complete this checklist by entering the page numbers from your manuscript where readers will find each of the items listed below.

Your article may not currently address all the items on the checklist. Please modify your text to include the missing information. If you are certain that an item does not apply, please write "n/a" and provide a short explanation.

Upload your completed checklist as an extra file when you submit to a journal.

In your methods section, say that you used the PRISMA-Preporting guidelines, and cite them as:

Moher D, Shamseer L, Clarke M, Ghersi D, Liberati A, Petticrew M, Shekelle P, Stewart LA. Preferred Reporting Items for Systematic Review and Meta-Analysis Protocols (PRISMA-P) 2015 statement. Syst Rev. 2015;4(1):1.

For peer review only

		Reporting Item	Page Number
**Title**			
Identification	#1a	Identify the report as a protocol of a systematic review	1
Update	#1b	If the protocol is for an update of a previous systematic review, identify as such	n/a
**Registration**			
	#2	If registered, provide the name of the registry (such as PROSPERO) and registration number	2
**Authors**			
Contact	#3a	Provide name, institutional affiliation, e-mail address of all protocol authors; provide physical mailing address of corresponding author	1
Contribution	#3b	Describe contributions of protocol authors and identify the guarantor of the review	15
**Amendments**			

	#4	If the protocol represents an amendment of a previously completed or published protocol, identify as such and list changes; otherwise, state plan for documenting important protocol amendments	n/a
**Support**			
Sources	#5a	Indicate sources of financial or other support for the review	16
Sponsor	#5b	Provide name for the review funder and / or sponsor	16
Role of sponsor or funder	#5c	Describe roles of funder(s), sponsor(s), and / or institution(s), if any, in developing the protocol	n/a
**Introduction**			
Rationale	#6	Describe the rationale for the review in the context of what is already known	2-3
Objectives	#7	Provide an explicit statement of the question(s) the review will address with reference to participants, interventions, comparators, and outcomes (PICO)	3-7
**Methods**			
Eligibility criteria	#8	Specify the study characteristics (such as PICO, study design, setting, time frame) and report characteristics (such as years considered, language, publication status) to be used as criteria for eligibility for the review	8-9
Information sources	#9	Describe all intended information sources (such as electronic databases, contact with study authors, trial registers or other grey literature sources) with planned dates of coverage	8
Search strategy	#10	Present draft of search strategy to be used for at least one electronic database, including planned limits, such that it could be repeated	8
Study records - data management	#11a	Describe the mechanism(s) that will be used to manage records and data throughout the review	10-11
Study records - selection process	#11b	State the process that will be used for selecting studies (such as two independent reviewers) through each phase of the review (that is, screening, eligibility and inclusion in meta-analysis)	8-9
Study records - data collection process	#11c	Describe planned method of extracting data from reports (such as piloting forms, done independently, in duplicate), any processes for	10-11

For peer review only

		obtaining and confirming data from investigators	
Data items	#12	List and define all variables for which data will be sought (such as PICO items, funding sources), any pre-planned data assumptions and simplifications	4-7
Outcomes and prioritization	#13	List and define all outcomes for which data will be sought, including prioritization of main and additional outcomes, with rationale	4-7
Risk of bias in individual studies	#14	Describe anticipated methods for assessing risk of bias of individual studies, including whether this will be done at the outcome or study level, or both; state how this information will be used in data synthesis	13-14
Data synthesis	#15a	Describe criteria under which study data will be quantitatively synthesised	11-15
Data synthesis	#15b	If data are appropriate for quantitative synthesis, describe planned summary measures, methods of handling data and methods of combining data from studies, including any planned exploration of consistency (such as I2, Kendall's τ)	11-15
Data synthesis	#15c	Describe any proposed additional analyses (such as sensitivity or subgroup analyses, meta-regression)	14-15
Data synthesis	#15d	If quantitative synthesis is not appropriate, describe the type of summary planned	11
Meta-bias(es)	#16	Specify any planned assessment of meta-bias(es) (such as publication bias across studies, selective reporting within studies)	13-14
Confidence in cumulative evidence	#17	Describe how the strength of the body of evidence will be assessed (such as GRADE)	14

The PRISMA-P checklist is distributed under the terms of the Creative Commons Attribution License CC-BY 4.0. This checklist was completed on 23. September 2019 using https://www.goodreports.org/, a tool made by the EQUATOR Network in collaboration with Penelope.ai

For peer review only
